# Effect of Fusion to the LTB Carrier Protein on Coronavirus Spike Protein Vaccine Candidates Produced in Maize

**DOI:** 10.3390/v17010007

**Published:** 2024-12-24

**Authors:** Erin Egelkrout, Magdalena Maj, Rodrigo Manjarin, Gina Fake, Muneaki Watanabe, Jenna Williams, Nate Blanchard, John Walker, Celine Hayden, John Howard

**Affiliations:** 1 Applied Biotechnology Institute, California Polytechnic Tech Park, San Luis Obispo, CA 93407, USA; erinegelkrout@appliedbiotech.org (E.E.); gfake@appliedbiotech.org (G.F.); mwatanabe@appliedbiotech.org (M.W.);; 2 Department of Biological Sciences, California Polytechnic State University, San Luis Obispo, CA 93407, USA; mmaj@calpoly.edu (M.M.);; 3 Department of Animal Sciences, California Polytechnic State University, San Luis Obispo, CA 93407, USA; rmanjari@calpoly.edu; 4 Department of Statistics, California Polytechnic State University, San Luis Obispo, CA 93407, USA

**Keywords:** PEDV, SARS-CoV-2, spike protein, oral delivery, plant-produced vaccines, maize, trimer

## Abstract

Coronaviruses continue to disrupt health and economic productivity worldwide. Porcine epidemic diarrhea virus (PEDV) is a devastating swine disease and SARS-CoV-2 is the latest coronavirus to infect the human population. Both viruses display a similar spike protein on the surface that is a target of vaccine development. Despite the availability of commercial vaccines for both viruses, there is still a high occurrence of infections and a great need for enhanced efficacy and lower costs. We previously produced the PEDV spike protein (S) using transgenic maize, enabling a low-cost supply of the vaccine candidate. In this study, we (1) test orally delivered PEDV vaccine candidates in pigs to optimize the mucosal immune response; (2) generate the SARS-CoV-2 S1 protein in maize; and (3) perform structural characterization of the S1 protein for PEDV and SARS-CoV-2. We demonstrated high expression levels in maize of the S1 subunit of the SARS-CoV-2 spike protein, both with and without fusion to the heat-labile enterotoxin B (LTB) subunit. We found that the LTB fusion protein from both coronaviruses preferentially assembles into higher molecular weight multimers, consistent with the formation of trimers. For PEDV, administering the spike protein fused to LTB to young pigs elicited a higher level of mucosal IgAs compared to maize grain containing the S1 protein alone or controls. This suggests that fusing the coronavirus spike protein with LTB may provide better protection.

## 1. Introduction

Viruses in the Coronaviridae family can cause widespread disease in both humans and animals. In one example relevant to livestock, porcine epidemic diarrhea virus (PEDV), a member of the alphacoronavirus genus, is a highly contagious enteric disease in swine that causes substantial losses in farming across much of Asia and North America. Newborn piglets, upon infection with PEDV, can suffer a mortality rate of up to 100% within 7 days after birth [1]. The conditionally approved injected vaccines for PEDV are not fully effective, induce little or no antibody (IgA) secretion at the mucosa level in pigs, and require cold-chain and labor-intensive parenteral administration [2,3]. An oral vaccine absorbed in the gastrointestinal tract may provide mucosal protection while being safe to administer to larger pig populations at minimal cost and labor, and without the risk of horizontal transmission.

More recently, SARS-CoV-2, a betacoronavirus that causes COVID-19, has had a devastating impact on global health and the economy, causing over 7 million deaths worldwide since its emergence in late 2019 [4]. This has led to an unprecedented effort by many organizations worldwide to develop vaccines, resulting in the approval of several different vaccines in record time. While these vaccines have significantly slowed the spread of SARS-CoV-2, better approaches to rapidly addressing this pandemic (or future pandemics) are needed. Current vaccines have many hurdles, including costs, the requirement for cold-chain storage and transport, and administration using sterile needles via trained personnel. Other impediments to vaccination include concerns regarding vaccine injection safety, vaccine adjuvants, fear of needles, and other factors that contribute to vaccine hesitancy. There is also a subset of the population who respond poorly to vaccination, remaining vulnerable to the disease. This leaves millions of people at risk as the virus continues to spread.

Although many vaccines are based on killed or live-attenuated pathogens, subunit vaccines comprising only key protein components (s) of the pathogen have the advantage of safety because they do not contain nucleic acid and cannot replicate. Many examples of subunit vaccines, such as vaccines for influenza and hepatitis B, are in widespread use. Animal alphacoronaviruses, such as (PEDV) and *transmissible gastroenteritis virus* (TGEV), as well as betacoronaviruses, such as SARS-CoV-1 and SARS-CoV-2, display a similar spike glycoprotein on the surface of the virus. This protein has been the primary antigen target for the current injectable vaccines against SARS-CoV-2. However, production systems, such as mammalian cell cultures for these proteins, along with the requirement for post-translational modifications, make these vaccines relatively expensive.

Expression in plants is one promising approach in the production of antigens for subunit vaccines. There is a precedent for the production and administration of vaccine candidates eliciting relevant immune responses for both PEDV and SARS-CoV-2 using plants [5,6,7,8,9]. The SARS-CoV-2 S protein receptor binding domain (RBD) was recently produced in tobacco [10,11,12,13]. Plant-produced RBD elicits a neutralizing response in mice and non-human primates. In a significant step for the development of plant-based vaccines, a plant-produced vaccine for SARS-CoV-2 from Medicago and Glaxo Smith-Kline was recently approved for use in Canada [14]. However, production in tobacco still requires immediate use of plant material, purification of the protein, and traditional parenteral administration.

Most vaccines approved thus far require parenteral administration and, while protecting against the most severe symptoms, have had limited efficacy in preventing infection due to their poor mucosal immune response. The development of vaccines via mucosal administration can help address this. SARS-CoV-2 vaccines via mucosal delivery were recently approved in China and a few other countries [15], and numerous others are in preclinical development [16,17,18]. The development of thermostable oral vaccines in a simpler formulation that can be easily administered to humans or incorporated into livestock feed would greatly facilitate the delivery of vaccines throughout the world.

In addition, in order to vaccinate the global population or massive numbers of livestock in a short period of time, novel production systems and a simpler means of administration will be required. In the case of SARS-CoV-2 vaccines, manufacturers face the unprecedented challenge of producing and distributing billions of doses to supply the global population. Therefore, despite encouraging progress, immunization for the world population will take years with the current vaccines. The widespread distribution of the virus, potential spread due to asymptomatic infection, the inability to rapidly immunize the world population, and survival of the virus in animal reserves make this virus likely to continue circulating for a longer time, thus increasing the chances of further mutations and the emergence of additional variants.

We developed a system for the production of subunit vaccines in transgenic maize that enables the production of high levels of antigens with a rapid scale-up at a relatively low cost, as well as the potential for oral administration without purification. Expression in maize grain also provides inherent bioencapsulation that stabilizes the protein in the digestive tract and enables storage and transport at ambient temperatures. We previously demonstrated the proof-of-concept for protein expression in maize and protection from challenges in animal studies using this system for the two porcine coronaviruses mentioned above, PEDV [19,20] and TGEV [21].

One major objective of the current study is to extend our previous work, demonstrating the protective efficacy of a low-cost prototype maize-produced PEDV spike protein. The spike proteins from alphacoronaviruses and betacoronaviruses have a common feature: the formation of a trimer that enhances immunogenicity. Moreover, SARS-CoV-2 vaccines include stabilizing mutations to increase the proportion of trimers in subunit vaccine production [22]. The use of stabilizing mutations or fusion to heterologous trimerization peptides has greatly facilitated the development of efficacious vaccines against SARS-CoV-2 and other viruses. To address both current and future disease outbreaks, developing additional approaches to stabilize trimer formation would be useful. Such strategies could enhance both human and veterinary subunit vaccines for protection against coronaviruses.

Vaccination to elicit anti-S antibodies against PEDV has been a key objective for the prevention and control of PEDV [2,23,24,25]. We previously described proof-of-concept studies for the efficacy of an orally delivered vaccine based on PEDV S1 targeted to the endoplasmic reticulum. To test for the increased efficacy of different constructs, we also prepared and transformed into maize a gene fusing the PEDV-S1 with the carrier protein, the B subunit of the heat-labile toxin of enterotoxigenic *E. coli* (LTB). LTB is used to increase the efficacy of vaccine candidates by binding to gangliosides present on cell surfaces, creating additional points of recognition for extracellular molecules that can enhance the immune response upon oral administration. For PEDV, one study has shown that the fusion of the core-neutralizing epitope (COE) with LTB retains the ability to bind GM1-gangliosides [26]. Another study used LTB as a separate adjuvant with injected PEDV-S1—the authors saw an increase in serum IgG and neutralizing antibodies but not fecal IgA [27]. Here, we demonstrated an increase in fecal IgA upon oral administration of the fusion of PEDV S1-LTB. We tested the effect of fusion to LTB on the formation of higher molecular weight multimers of the S1 protein.

As part of the second major objective of this work, we demonstrated the expression of the SARS-CoV-2 S1 protein in maize, derived from an early strain, featuring different subcellular localizations and fusion to carrier proteins, including LTB. Again, we evaluated the effect of fusion to LTB on the formation of higher molecular weight multimers of the S1 protein.

## 2. Materials and Methods

### 2.1. Production of Maize Expressing PEDV S1 Antigen

Transgenic maize lines expressing high levels of the S1 protein were previously created [19]. The PDC construct contained the S1 protein and the PDK construct contained the S1 protein fused to the carrier protein, LTB (the non-toxic subunit B of the heat-labile toxin from *E. coli*). Expression of the S1 coding region was directed to the corn embryo. Plants from both lines were grown to obtain grain for the animal study.

### 2.2. Western Blot Analysis–PEDV

Grain was evaluated using Western blot to determine the S protein content. One hundred mg samples of PDC and PDK corn flour were extracted with 1 mL of 1X PBS, loaded onto a 4–12% Bis-Tris gel (Invitrogen, Waltham MA, USA, no. NP0336), and transferred to the PVDF membrane via iBlot. The blot was incubated with rabbit-anti-PEDV S1 (Pacific Immunology, Ramona, CA, USA, custom-made) overnight at a dilution of 1:4000 or with anti-LTB (Bethyl Labs, Montgomery, TX, USA, custom-made) overnight at a dilution of 1:4000, and developed with an anti-rabbit-alkaline phosphatase conjugate at a dilution of 1:2000 (Jackson ImmunoResearch, West Grove, PA, USA, no. 111-055-003) and a BCIP-NBT liquid substrate (Sigma, St Louis, MO, USA, no. B1911). The COE standard synthesized by GenScript [19] was loaded as a positive control and the concentration of S1 was estimated by comparing it to the standard. The final S1 antigen content was estimated from Western blot images (*n* = 7–8 samples per maize line).

### 2.3. Size Exclusion Chromatography–PEDV

Ground maize flour samples (100 mg) from PDC and PDK were extracted in 1 mL 1X PBS. The extract supernatant was filtered through Whatman no. 1 paper and loaded onto a calibrated Superdex 200 (Cytiva, Marlborough, MA, USA, no. 17104301) size exclusion chromatography column (Bio-Rad, Hercules, CA, USA, no. 7371022) with a fractionation range (Mr) of 10,000 to 600,000 to separate molecules by size. A flow rate of 1 mL/min, controlled by an Econo Pump (Bio-Rad no. 7318300), a column length of 20 cm, a particle pore size of 34 um, and protein standards (Cytiva no. 28-4038-42), including Blue Dextran, Ovalbumin, Conalbumin, Aldolase, Ferritin, and Thyroglobulin, were used. Fifty-microliter fractions were collected from the column and stored at 2–8 °C for analysis by ELISA as described below.

### 2.4. ELISA–PEDV S1 Protein in Size Exclusion Chromatography Samples

Nunc-Immuno MaxiSorp microtiter plate wells were coated with 0.3 ug of anti-PEDV S1 (Bethyl Laboratories, Montgomery, TX, USA, custom) and stored at 2–8 °C overnight. The plate was washed with 1X PBS + 0.05% Tween20 (PBST) between subsequent steps and all incubations lasted for 1 h at 37 °C. After washing, the wells were blocked with a 2% bovine serum albumin (BSA) in PBST. Size exclusion chromatography (SEC) fractions were loaded alongside a PEDV S1 standard (GenScript, Piscataway, NJ, USA, custom) diluted in 0.1% BSA in PBST to create a standard curve. Biotinylated anti-PEDV S1 (Bethyl Laboratories, custom-made) was diluted 1:3000 and added to the plate followed by a streptavidin alkaline phosphatase conjugate diluted 1:1000 (Jackson ImmunoResearch no. 016-050-084). After substrate incubation (SeraCare, Milford, MA, USA, no. 5120-0059), the plate was read at 620 nm. The concentration of PEDV S1 in the chromatography fractions was calculated using the standard curve.

### 2.5. Preparation of the Corn Material for Animal Trials

PDC and PDK germ were dried to a moisture content of less than 12% and ground on a Glen Mills grinder (model EG43, Clifton, NJ, USA) to obtain corn meal, such that >80% of the material could pass through a 20-mesh screen. Individual bags of 50–100 g of corn meal were packaged and labeled with a letter and color code representing the different treatments. Each bag contained 900 µg (PDC) or 300 µg (PDK) of the antigen. The code was not shared with those conducting the animal trial. Commodity corn was used for the control animals.

### 2.6. Animals and Experimental Design

The animal trial was conducted in accordance with the Institutional Animal Care and Use Committee of California State University (no. 1611), and the National Research Council Guide for the Care and Use of Laboratory Animals. Twenty 19- to 23-day-old male (M) and female (F) Landrace pigs were moved into a temperature-controlled room with a 12:12-h light-dark cycle. Each animal was given two ear tags to be uniquely identified. Pigs were housed in groups of five animals in 1.5 × 1.5-m pens, balanced for sex and weight. All animals used in this study were determined to be free from PEDV via real-time RT-PCR analysis of fecal material using Real PCR PEDV/PDCoV Multiplex RNA Mix (IDEXX, Westbrook, ME, USA, no. 99-56450) and by a serum-neutralizing assay. The latter was a PEDV cytometry-based high-throughput neutralization test (HTNT) assay performed by the Iowa State University Veterinary Diagnostic Laboratory (ISU VDL, Ames, IA, USA).

After 3 days of acclimation, pens were randomly assigned to receive one of four treatments (*n* = 5 per treatment) (Table 1). The first day of the vaccine administration was considered as day 0 of the study. Pigs received boosts three times during the study. To ensure the complete uptake of the material in oral vaccine groups, animals were fasted overnight before feeding and then returned to their normal diet an hour after vaccine administration. Each animal was individually fed with one bag of maize meal per day for 3 consecutive days and consumed the full dose of maize material offered. Animals in the positive control group were intramuscularly injected with 2 mL of a commercially available PEDV vaccine (Zoetis, Parsippany, NJ, USA) containing an undisclosed concentration of killed virus, polysorbate 80, Merthiolate, gentamicin, 4–6% aluminum hydroxide, 1% mineral oil, and <5% of sorbitan oleate. Pig weight was recorded on the first and last day of the animal trial, as well as each time the treatment was administered.

All pigs were observed daily for general health. Blood samples were collected in serum collection tubes on days 0, 20, 34, 48, and 62. Fecal matter samples were collected in sterile tubes on days 0, 21, 28, 30, 32, 42, 44, 46, 56, 58, 60, and 62. All samples were subsequently frozen at −20 °C until the analysis was performed. Animals were euthanized on day 62 using an intramuscular injection of 4 mg/kg tiletamine and zolazepam (Zoetis), followed by an intracardiac injection of 0.4 mL/kg pentobarbital sodium (Schering-Plough, Rahway, NJ, USA). Each animal was tested for ocular reflexes and observed for respiratory movements.

### 2.7. PEDV-Neutralizing Antibodies

Neutralizing antibodies for PEDV were determined via the fluorescent focus neutralization assay performed at the South Dakota State University Diagnostics Laboratory. In brief, Vero-76 cells were seeded onto 96-well microplates and cultured for 3–4 days. Serum samples were added to the cells in serial 1:2 dilutions and PEDV virus stock was added at approximately 100 focus-forming units (FFU)/well. After overnight incubation in a minimum essential medium with trypsin, cells were fixed via the addition of 80% acetone. PEDV-specific monoclonal antibody SD6-29 conjugated to FITC was added and binding was assessed using a fluorescent microscope. A sample was considered positive if 90% inhibition of fluorescent foci was observed and the titer was reported as the highest dilution that had ≥ 90% inhibition.

### 2.8. Anti-IgA ELISA–PEDV

An ELISA was developed to measure the level of anti-PEDV IgAs in porcine fecal samples based on previous studies [28,29,30]. An ELISA plate (Thermo Fisher no. 12-565-136) was coated with 100 µL of PEDV S1 protein (GenScript, custom) at 0.100 µg/well in 0.05 M NaHCO_3_ coating buffer. The plate was stored overnight at 4 °C on a flat surface to ensure effective coating. About 50 mg (wet weight) of fecal samples were extracted with a cold extraction buffer (0.01 M PBS pH 7.4, 0.5% Tween 20, and 0.05% sodium azide). Samples were homogenized for 1 min, centrifuged in the cold at 1500× *g* for 20 min, and the supernatant was transferred to a sterile microcentrifuge tube containing 2 µL of a protease inhibitor cocktail (Pierce, Waltham MA, USA, no. A32965). Samples were vortexed and centrifuged in the cold at 10,000× *g* for 10 min. A pre-coated ELISA plate was washed 4 times with PBST, loaded with 100 µL of fecal extraction samples, and incubated at 37 °C for 2 h. After washing 4 times with PBST, 100 µL of the secondary antibody HRP-conjugated goat anti-pig IgA (Abcam, Cambridge, UK, no. ab112746) at a 1:5000 ratio was added. The plate was incubated at 28 °C for 1 h and washed 4 times with PBST; 100 µL of Sure Blue Reserve TMB peroxidase substrate (SeraCare no. 5150-0083) was added and it was incubated for 8 min in a dark cabinet at RT. After incubation with the substrate, 100 µL of TMB stop solution (SeraCare no. 5150-0021) was added and the plate was read on the SpectraMax Plus 384 (San Jose, CA, USA) at 450 nm within 5 min of adding the stop solution.

### 2.9. Statistical Analysis–PEDV Animal Trial and IgA ELISA

IgA in fecal samples was analyzed via a two-way ANOVA using a mixed model in SAS 9.2 (PROC MIXED; SAS Institute Inc., Cary, NC, USA) that included treatment x day as a fixed effect. Serum levels of neutralizing antibodies on d 62 were analyzed via a one-way ANOVA using a mixed model that included treatment as a fixed effect. Pig growth was analyzed via a two-way ANOVA using a mixed model that included treatment x day as a fixed effect, as well as a repeated measurement statement with the day as a repeated factor and the pig as a subject. The normality of residuals and the presence of outliers were assessed with PROC UNIVARIATE (SAS). Non-normally distributed parameters were power-transformed by a parameter φ whose optimal value was estimated using the maximum likelihood method. Data are presented as mean ± SD. Multiple comparisons were corrected with the Tukey post hoc test and significant effects were considered at *p* ≤ 0.05.

### 2.10. Construct Preparation–SARS-CoV-2

**Figure 1:**
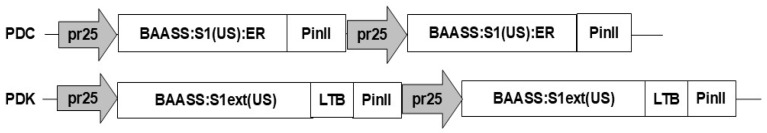
PEDV construct diagrams. Diagrams for each of the constructs prepared and tested are shown. PDC and PDK are two DNA constructs used for expressing the S1 protein from PEDV. Promoter pr25 is a 3 Kb fragment of the maize globulin 1 promoter (*glb1*). BAASS indicates a cell-wall targeting sequence, while ER indicates signaling sequences for the endoplasmic reticulum. LTB indicates fusion to the carrier protein, the B subunit of the heat-labile toxin of enterotoxigenic *E. coli*. PinII is the terminator sequence from potato proteinase inhibitor II.

The sequence of the spike protein from the original Wuhan-Hu-1 strain (GenBank, Bethesda, MD, USA, NC_045512.2) was optimized for maize codon usage. The nucleotide sequence of the coding region was outsourced for commercial gene synthesis by GenScript. Three different constructs were prepared (Figure 1) with varying subcellular targets and fusions. The native signal sequence was replaced with a barley alpha-amylase signal sequence (BAASS) in all constructs: COA, COB, and COD. In the COB construct, the amino acid sequence KDEL was added to the carboxy terminus to target it to the ER. In the COD construct, the LTB peptide was added to the carboxy terminus. In all constructs, the S1 coding region (aa 23–684) was synthesized for transfer to the maize transformation vector pSB11 using a NcoI site overlapping the initiating ATG and a PacI restriction site in the terminator region to insert the coding region downstream of the promoter. All constructs incorporated the S1 coding region under the control of a synthetic promoter derived from the maize globulin-1 gene, pr44, which targets expression in the corn embryo. This promoter was an engineered version of the previously described pr25 promoter that contained two extra copies of the 5′ region of pr25 [31]. Each transcription unit also incorporated the terminator from potato proteinase inhibitor II. Subcellular localization and terminator sequences were as previously described [31].

### 2.11. Plant Transformation–SARS-CoV-2

Maize transformation was carried out as previously described with modifications [32]. In brief, the constructs were transferred into the LBA4404 *Agrobacterium* strain containing the vector pSB1 using a triparental mating procedure [33]. The cointegrate DNA was then electroporated into the *Agrobacterium tumefaciens* strain EHA101 [34]. HiII maize embryos 1.5 to 3 mm in length were mixed with *A. tumefaciens* EHA101, harboring the appropriate vector for transformation [32]. Plants from events selected on the bialaphos were grown to maturity in the greenhouse and pollinated with HiII to produce the T1 generation seed. A total of 128 independent transformation events (at least 10 per construct) and 112 plants (at least 21 per construct) were obtained for the three different constructs (COA, COB, and COD).

### 2.12. Western Blot Analysis–SARS-CoV-2

Proteins were extracted from the ground maize seed with PBS + 1% SDS, loaded onto a 4–12% bis-tris gel (Thermo Fisher Scientific, Waltham, MA, USA, no. NP0336), and transferred to the PVDF membrane via iBlot. The blot was incubated in an anti-SARS-CoV-2 spike protein receptor binding domain (RBD) (Sino Biological, Houston, TX, USA, no. 40592-T62) overnight at a dilution of 1:2000, or with anti-LTB (Bethyl Labs, custom-made) at a dilution of 1:4000. It was developed with an anti-rabbit-alkaline phosphatase conjugate at a dilution of 1:2000 (Jackson ImmunoResearch no. 111-055-003) and BCIP-NBT liquid substrate (Sigma no. B1911). The positive S1 protein control was 10 ng of standard from Sino Biological (cat. no. 40591-V08H), and the concentration of S1 was estimated using this standard.

### 2.13. ELISA Analysis–SARS-CoV-2 Spike Protein

Individual seeds from SARS-CoV-2 transgenic maize were pulverized and extracted in PBS + 0.05% Tween-20 (PBST). Nunc-Immuno MaxiSorp 96-well microtiter plates were coated with 0.3 µg of SARS-CoV-2 (2019-nCoV) Spike Neutralizing Antibody, Rabbit Mab (Sino Biological no. 40592-R118) overnight. After washing, plates were blocked for 1 h in 2% BSA in PBST. Seed extracts were diluted 1:250 in 0.1% BSA in PBST. SARS-CoV-2 (2019-nCoV) Spike S1 (K417N, E484K, N501Y, D614G)-His (Sino Biological no. 40591-V08H10) was used as the recombinant protein standard. SARS-CoV-2 (2019-nCoV) Spike Neutralizing Antibody, Mouse Mab (HRP) (Sino Biological no. 40591-MM43-H) was used as the detection antibody. After washing, 100 µL of TMB SureBlue Reserve peroxidase substrate was added (KPL, Milford, MA, USA, no. 53-00-03). After incubation with the substrate, 100 µL of TMB stop solution (KPL no. 50-85-05) was added and the plate was read at 450 nm. The concentration of SARS-CoV-2 S1 in the seed extracts was calculated using the standard curve.

### 2.14. Size Exclusion Chromatography/(Fast Protein Liquid Chromatography (FPLC))–SARS-CoV-2 S1

Ground maize flour samples (100 mg) of COB and COD were extracted in 1 mL 1X PBS. The extract supernatant was filtered through Whatman no. 1 paper and loaded onto a calibrated Superdex 200 (Cytiva, Marlborough, MA, USA, no. 17104301) size exclusion chromatography XK 16/20 column (Cytiva no. 28988937). A flow rate of 1 ml/min, 45 mL of packed resin, a particle pore size of 34 um, and a protein standard mix (Sigma no. MWGF200) including blue dextran, b-amylase, alcohol dehydrogenase, albumin, carbonic anhydrase, and cytochrome c were used. Column fraction samples of 0.5 mL were collected and analyzed for SARS-CoV-2 S1 via ELISA as described above.

### 2.15. Statistical Analysis–SARS-CoV-2 Spike Protein

The data were analyzed using a linear mixed model in both JMP version 16.2 and R version 4.2.2 (lme4 package version 1.1-31). Both programs produced similar results. The initial response variable was the percentage of total soluble protein (TSP) of individual seeds. The predictor variables were the construct, plant, and ear. The construct was a fixed effect with levels A, B, and D. The plant and ear were random effects with the plant nested within the construct and the ear nested within both the plant and construct. The residual TSP percentages for seeds from the same ear and plant were assumed to be correlated, but uncorrelated for seeds from different plants.

As the initial model, using percent TSP as the response variable had highly skewed residuals and very unequal residual variance between the three constructs. Percent TSP was transformed using the formula log_2_ (‘percent TSP’ + 1). Adding 1 to each observation was required because of the zero percent TSP values present in the data for which the log would have been undefined. The final model used the transformed percent TSP with the same predictor variables as the initial model. In this model, the residuals were less skewed and the residual variances were more similar between constructs.

The final model showed extremely strong evidence of differences in the mean transformed percent TSP between some of the three constructs (*p*-value < 0.0001). Tukey’s method was used to compare all pairs of constructs. Construct A had a higher mean transformed percent TSP than Construct B or Construct D. Comparisons between Constructs B and D were unable to detect differences. Constructs that shared the same lower-case letter were statistically indistinguishable at the 5% overall significance level using Tukey pairwise comparisons. A similar comparison was performed for the top ten seeds.

## 3. Results

### 3.1. LTB Fusion Increases the Formation of Spike Protein Multimers for PEDV

**Figure 2:**
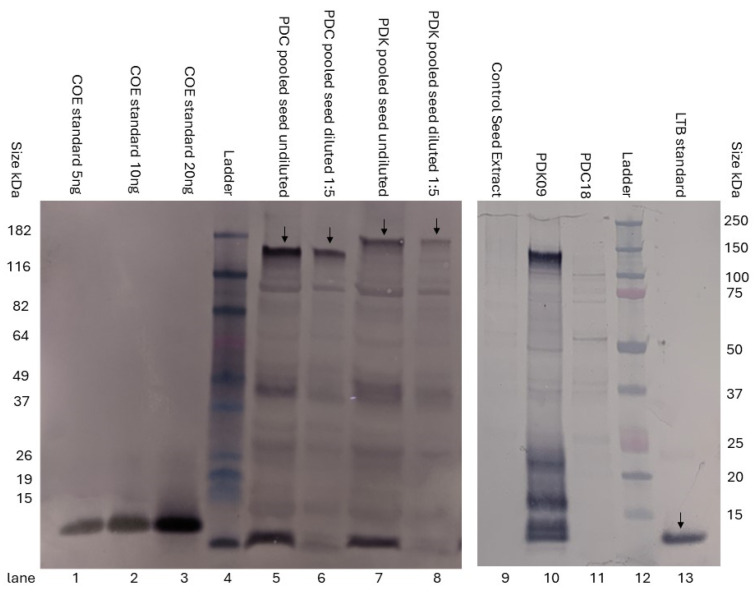
Western blot analysis of PEDV S1 expression. The pooled seed for the indicated plant was ground and extracted in 1X PBS + 1% SDS for selected independent transformation events for constructs PDC (lanes 5–6) and PDK (lanes 7–8). The protein was transferred to the PVDF membrane and incubated with polyclonal rabbit antibody raised to the COE domain of PEDV S1 (**left panel**). The COE recombinant protein produced by GenScript is shown as a positive control in lanes 1–3. Extracts from constructs PDC (lane 11) and PDK (lane 10) were probed with a custom antibody to LTB (**right panel**).

**Figure 3:**
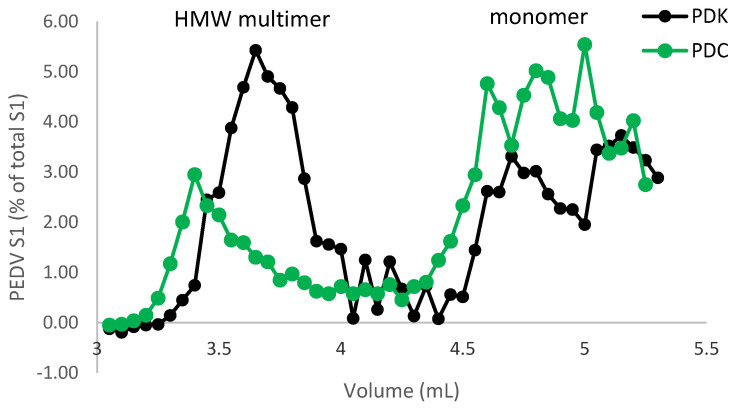
SEC and FPLC analysis of the PEDV spike protein with and without the LTB tag. Maize lines expressing PEDV S1 (PDC) or S1:LTB fusion (PDK) were extracted and run on a size exclusion column. Fractions (0.5 mL) collected from the column were analyzed for PEDV S1 via ELISA. The S1:LTB showed a higher ratio of trimers to monomers compared to S1 alone.

The production of constructs for PEDV was previously described (Figure 1 and [19]). Figure 2 confirms the relative sizes of constructs PDC and PDK as well as the recognition of the LTB tag via western blot. To examine the effects of the LTB tag on potential trimer formation, size exclusion chromatography (followed by ELISA for PEDV S1 levels in the fractions) was used to compare the levels of different molecular weight complexes in extracts from maize material for constructs PDC and PDK (Figure 3). Both constructs showed the presence of the S1 protein, with both monomers consistently eluting at approximately 70–90 kDa and higher molecular weight complexes eluting at approximately 400–600 kDa. However, the PDK S1:LTB extracts showed more than a two-fold increase in the level of high molecular weight S1 (potential trimer formation) over the construct with S1 alone based on the ELISA detection (based on an estimate of the area under the relevant curves). The column was calibrated with protein standards and consistently repeated for retention time and volume.

### 3.2. Oral Administration of Maize Expressing Fusion of Spike Antigen-LTB Carrier Protein Elicits Mucosal Anti-PEDV IgA Response in Pigs

**Figure 4:**
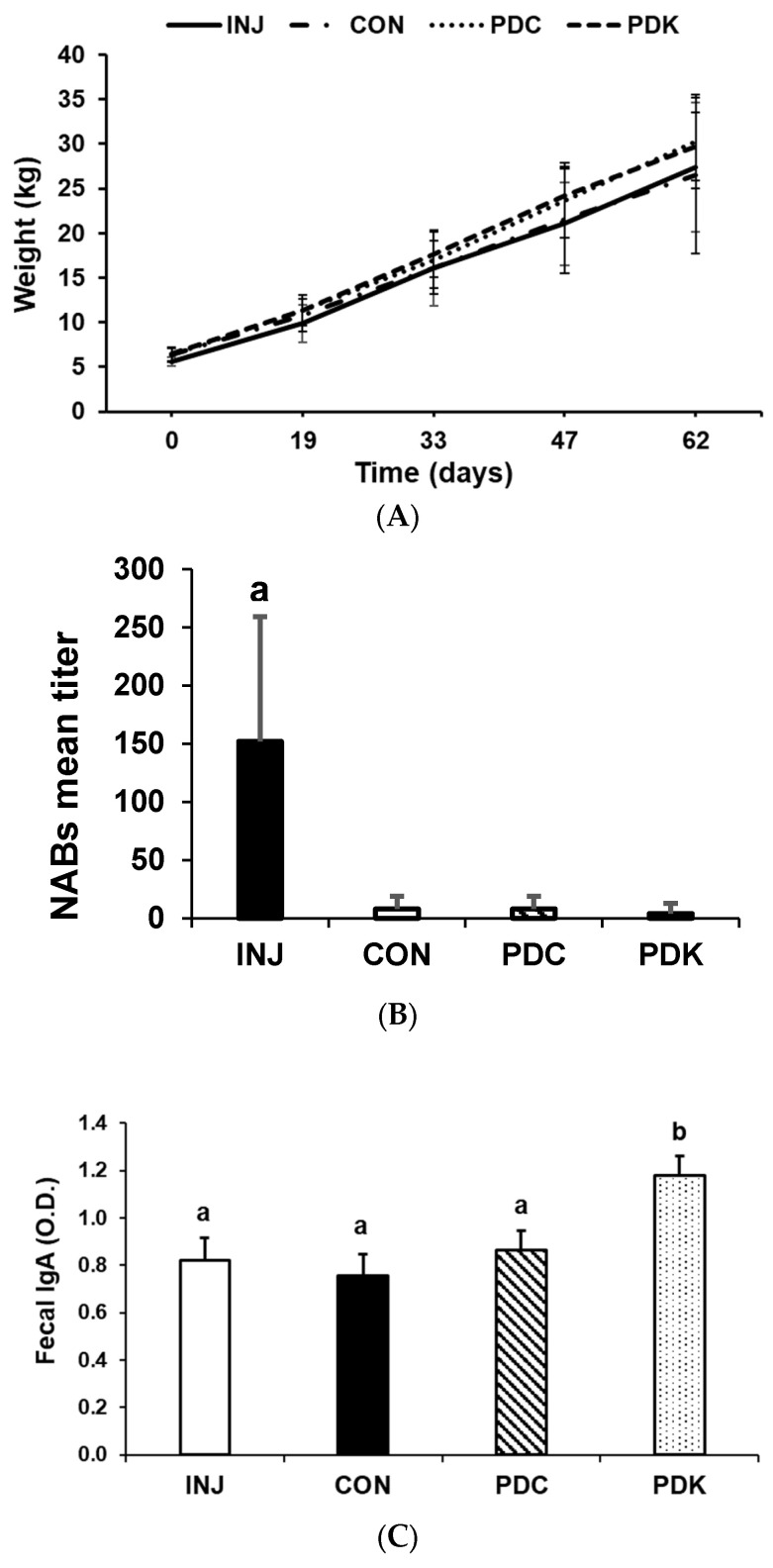
Pig trial results. Oral administration of maize expressing S1:LTB fusion elicits mucosal anti-PEDV IgA response in pigs. (**A**) Pig weight did not differ between the four treatment groups: pigs administered an injected PEDV vaccine (INJ; *n* = 5), a control group fed not-genetically modified corn (CON; *n* = 5), an oral PEDV vaccine candidate corn expressing PDC construct (PDC; *n* = 5), and an oral PEDV vaccine candidate corn expressing PDK construct (PDK; *n* = 5). (**B**) The level of neutralizing antibodies (NABs) in serum on the last day of the study (day 62) was higher in pigs administered injected PEDV vaccine boosters (INJ) compared to CON, PDC, and PDK groups. Results are presented as a titer that provided a positive result, with a dilution of 20-fold being the limit of detection for a positive sample. Values are mean ± SD. Values with different letters (a, b) *p* ≤ 0.05. (**C**) The level of mucosal anti-PEDV IgA was higher in pigs administered an oral PEDV vaccine candidate corn expressing PDK construct (PDK; *n* = 5) as compared to INJ, CON, and PDC.

Young pigs in all treatment groups (Table 1) were primed with one dose of a commercially available injected vaccine against PEDV. They were then boosted with (a) the parenterally administered commercial vaccine, or orally boosted with (b) control corn, (c) corn meal from the PDC construct (S1 alone), or (d) corn meal from the PDK construct (S1:LTB). Throughout the study, the pigs’ weight did not change between treatment groups (*p* ≤ 0.05) (Figure 4A) and we did not observe any health issues. Pigs injected multiple times (one primary and three boosts) with the anti-PEDV vaccine had significantly higher levels of neutralizing antibody (NAB) titers in sera on the last day of the study (day 62) compared to all other treatments (*p* ≤ 0.05; Figure 4B), while the CON, PDC, and PDK groups had barely detectable (if any) NABs. Because PEDV attacks mucosal barriers, we next evaluated whether the vaccine candidates triggered mucosal immune response. The levels of porcine fecal anti-PEDV IgAs were analyzed via ELISA. Only the animals administered the PDK vaccine candidate had significantly higher levels of anti-PEDV IgAs (*p* ≤ 0.05; Figure 4C) after the second boost compared to all other treatments despite the dose being much lower than the PDC line.

### 3.3. Molecular Constructs and Maize Transformation for the COVID-S1 Protein

**Figure 5:**
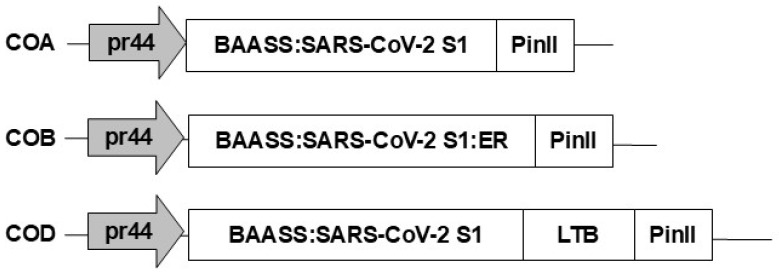
SARS-CoV-2 construct diagrams. Diagrams for each of the constructs prepared and tested are shown. COA, COB, and COD are three DNA constructs for expressing the S1 protein from SARS-CoV-2. Promoter pr44 is an embryo-preferred derivative of the maize globulin 1 (*glb1*) promoter with two additional copies of part of the *glb1* promoter. BAASS indicates a cell-wall targeting sequence, while ER indicates signaling sequences for the endoplasmic reticulum. LTB indicates fusion to the carrier protein, the B subunit of the heat-labile toxin of enterotoxigenic *E. coli*. PinII is the terminator sequence from potato proteinase inhibitor II.

Three different constructs were prepared to transform maize to produce maize lines expressing high levels of the SARS-CoV-2 spike protein from the original Wuhan–Human-1 strain for antigen production against SARS-CoV-2 (Figure 5). The constructs were designed based on our previous work, targeting two different subcellular locations and fusing to a carrier protein to maximize expression and potential immune response. All three constructs incorporated the S1 subunit (aa 23–684) under the control of the synthetic pr44 promoter based on the maize *globulin-1* promoter that targets expression in the embryo. The native signal sequence was replaced with the well-characterized BAASS for secretion with the default targeting to the cell wall in all three constructs. In the COB construct, the KDEL endoplasmic reticulum-targeting signal was added to the carboxy terminus for targeting the ER. In the COD construct, the LTB peptide was added to the carboxy terminus. These were used to construct expression vectors with the embryo-preferred promoter, coupled with an herbicide resistance gene, and then employed to transform corn as previously described [31,35].

### 3.4. Analysis of Expression in the T1 COVID-S1 Seed

**Figure 6:**
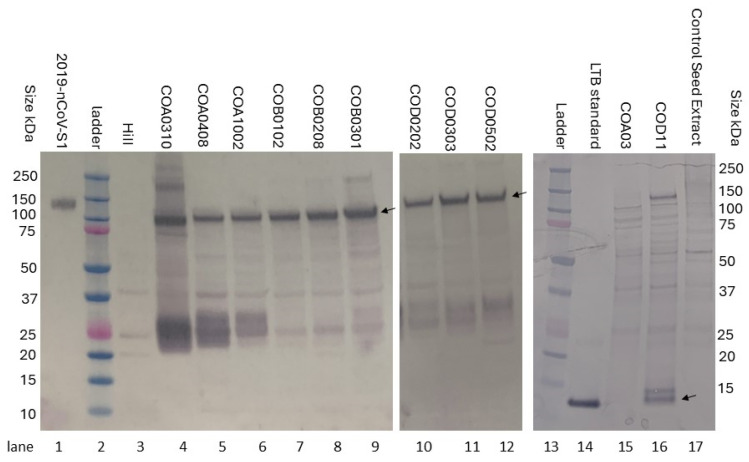
Western blot analysis of the SARS-CoV-2 S1 expression. The pooled seed for the indicated plant was ground and extracted in 1X PBS + 1% SDS for selected independent transformation events for constructing COA (lanes 4–6), COB (lanes 7–9), and COD (lanes 10–12) as well as untransformed maize seed (HiII, lane 3). The protein was transferred to the PVDF membrane and incubated with polyclonal rabbit antibody raised to the receptor binding domain (RBD) of the SARS-CoV-2 spike protein (**left** and **middle panels**). The SARS-CoV-2 S1 subunit recombinant protein produced in HEK293 cells (Sino Biological) is shown as a positive control in lane 1. The predicted size for the standard and maize-produced protein is 77 kDa. The bands for both standard and maize-produced S1 run at higher apparent molecular weights than predicted, possibly due to post-translational modifications such as glycosylation, as observed by the manufacturer of the standard. Extracts from constructs COA and COD were also probed with a custom antibody to LTB (**right panel**).

The expression of the S1 antigen was analyzed via western blot in crude maize extracts from pooled seed from selected independent transformation events from the three constructs. A commercially available polyclonal antibody against the SARS-CoV-2 spike protein or a custom antibody to LTB was used (Figure 6). A unique band, not present in the untransformed seed, was observed for all constructs. The predicted size of the SARS-CoV-2 (2019-nCoV) spike protein S1 subunit standard (Figure 6 lane 1) as well as the S1 subdomain produced in this project is approximately 77 kDa, based on the amino acid composition. The sizes of both standard and maize-produced recombinant protein bands migrate at higher molecular weights, ca. 100–140 kDa, compared to that predicted by the amino acid sequence, likely due to glycosylation, which can account for up to half the molecular weight of some viral surface proteins. Differences in glycosylation between mammalian cells and maize as well as the addition of a His tag to the standard may account for any difference in apparent size.

**Figure 7:**
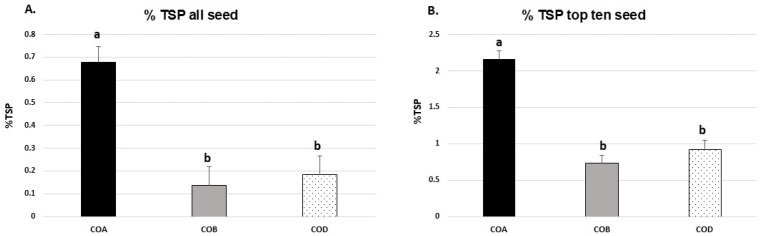
SARS CoV-2 S1 expression in T1 generation plants via ELISA. SARS CoV-2 S1 expression in all seeds (**A**), and the ten seeds with the highest levels of S1 expression (3B). The expression of SARS CoV-2 S1 as a percentage of TSP in the T1 seed is shown for constructs COA, COB, and COD. Values are means ± SD for all positive seeds for each construct (**A**) and for the ten highest expressing seeds (**B**). Letters (a, b) represent significant differences using Tukey’s pairwise comparison test at *α* = 0.05.

An ELISA assay was developed to quantify the level of S1 protein expression (Figure 6). Protein from six individual seeds from T1 generation plants (at least 19 plants per construct) for each construct and event was extracted and assayed for expression. The mean S1 protein expression level over all events indicated that expression in the COA construct at 0.68% TSP was higher than the other two constructs: COB (0.14%) and COD (0.19%) (*p* < 0.001) (Figure 7A). Typically, after the initial pollination of T0 generation plants, the highest expressing T1 generation lines are selectively back-crossed into commercially relevant elite lines with better yields in field conditions. Thus, as a more relevant indication of the potential accumulation levels, the average of the highest ten expressing seeds for each construct was also analyzed (Figure 7B). For the COA construct, an average of 3.73% tsp was observed. The process of back-crossing and optimization is currently ongoing and based on the calculation; our COA maize line potentially translates to/contains over 500 mg antigen per kg grain.

### 3.5. LTB Fusion Increases the Formation of Spike Protein Multimers for SARS-CoV-2

**Figure 8:**
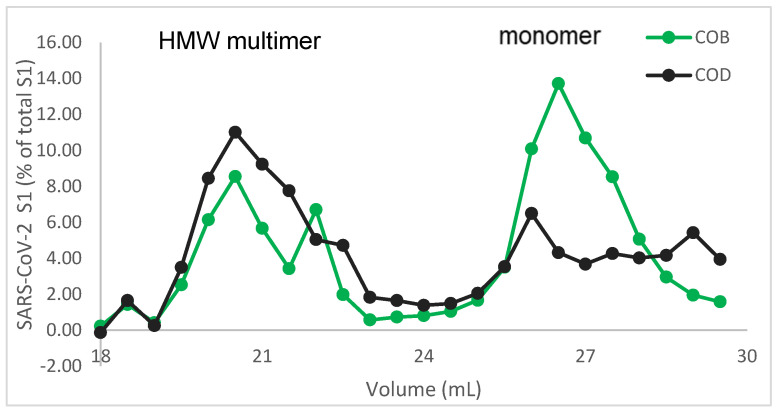
SEC and FPLC analysis of the SARS CoV-2 spike protein with and without the LTB tag. Maize lines expressing SARS-CoV-2 S1 (COB) or S1:LTB fusion (COD) were extracted and run on a size exclusion column. Fractions (0.5 mL) collected from the column were analyzed for SARS-CoV-2 S1 by ELISA. The S1:LTB showed a higher ratio of trimers to monomers compared to S1 alone.

To assess whether a similar effect to the PEDV spike protein was seen with the SARS-CoV-2 spike protein, extracts from constructs COB (S1 with no tag) and COD (S1 fused to LTB carrier) were separated by size exclusion chromatography, followed by ELISA (Figure 8). The COB construct was chosen for this purpose as a more relevant comparison to the COD construct than the COA construct for consistency with the PEDV work. Both constructs showed the presence of the S1 protein, consistent with both monomers and higher molecular weight complexes. In addition, a similar trend with the formation of higher levels of higher molecular weight complexes, consistent with trimers, was observed with the SARS-CoV-2 S1:LTB fusion. The FPLC was repeated several times to confirm the molecular size based on the protein standards with similar results.

## 4. Discussion

As viruses such as PEDV and SARS-CoV-2 continue to circulate, there is a need for novel production methods, high protective efficacy, and quick administration to enable mass vaccination of livestock and the world’s population, respectively. Production of viral antigens in transgenic maize enables high levels of expression, such that billions of doses can be rapidly scaled up and used as low-cost supplies for parenteral vaccines, or may enable the production of a thermostable oral vaccine. The spike proteins of both these viruses are lead targets for subunit vaccine development, and in both cases, form a homotrimer that may increase immunogenicity. For SARS-CoV-2 existing vaccines incorporate several stabilizing mutations to facilitate trimer formation in a prefusion state [36,37]. Al-though fusion to trimerization domains has been reported, there has been less research about comparable mutations for PEDV [9,38,39,40].

As the first step to developing coronavirus vaccines in maize, we previously produced the S1 subunit of PEDV in maize and demonstrated a proof of concept for the protection of pigs from the virus [20]. The LTB peptide, when fused to antigens, has shown an increase in immunogenicity, presumably due to its binding of gangliosides [41,42]. During the course of size exclusion characterization of the fusion protein via chromatography, we observed that the molecular weight of the S1:LTB protein was consistent with trimer formation in contrast to the S1 protein alone, where the dominant form was consistent with monomers. We demonstrated that the addition of the LTB tag to the PEDV spike protein enhances immunogenicity in juvenile pigs. While it was not our intent to describe the time course in detail, and we grouped time points to focus on the overall response, the mucosal immune response was highly transitory and could vary dramatically from day to day; at some time points, a larger 3-fold increase in fecal IgA in response to the LTB fusion was observed. Feeding corn containing the spike protein with no tag (construct PDC) did not result in a mucosal immune response in unchallenged pigs. This is consistent with previous studies, where an immune response was observed only after a challenge [19]. It should also be noted that although a neutralizing antibody response was not observed here, a neutralizing antibody response was observed after a challenge or in pregnant and lactating sows in previous work [19,20]. However, feeding corn containing the spike protein with fusion to LTB resulted in a robust fecal IgA response, suggesting that the S1:LTB peptide is more effective at eliciting a mucosal response in the absence of a viral challenge. It is not clear, however, if this is due to the ability of LTB to bind to gangliosides or the increased immunogenicity of the trimer. Future experiments in newborn piglets after a challenge will address the response to the addition of the LTB fusion in more detail and are likely to result in an increased neutralizing antibody and mucosal IgA response. While the data are consistent with the formation of trimers, the sizes of the higher molecular weight peaks are at higher molecular weights than predicted based on the amino acid sequence alone. Future work will be needed on full biochemical characterization to confirm whether the size of the HMW peak is due to post-translational modifications such as glycosylation, interactions between the LTB subunit, or the formation of other types of complexes. However, although LTB inherently forms a pentamer, there is less evidence for the role of LTB in the formation of multimers on fusion to a vaccine-candidate antigen. It is also unlikely that glycosylation can account for the large increase in the multimer size observed, and other types of aggregation might result in a wider distribution of sizes on chromatography relative to the peak observed. Nevertheless, as observed in other systems [43,44,45,46], fusing the LTB to peptide subunit vaccines produced in maize appears to be a good strategy to increase immunogenicity against other pathogens to increase the mucosal immune response.

In this work, antigen production for a subunit vaccine against SARS-CoV-2 was also established in maize expressing the spike protein from the virus responsible for COVID-19. The initial maize lines produced the protein at levels of 50–100 mg/kg of dried seed or an average of 3.73% TSP in the highest ten seeds for the COA construct. While the initial levels of expressions in the COB and COD constructs were lower than that in COA after quantitation via ELISA, optimization and selection in a back-cross program could increase antigens in these two constructs to levels comparable to those used in studies with other antigens. This will enable future studies to investigate the potential enhanced efficacy of the LTB fusion, as demonstrated with PEDV. The use of fusion to LTB in prototype SARS-CoV-2 vaccines has been described in a few studies but with very limited direct comparison to formulations without LTB [47,48]. In addition, as the stabilizing mutations for the mRNA1273 vaccine are in the S2 subunit not included in the recombinant protein described here, the addition of LTB may facilitate the formation of trimers with the smaller S1 subunit. Thus, it was of interest to compare the amounts of high molecular weight materials in constructs with different subcellular localizations and carrier protein fusions. Similar to the observation for PEDV, the addition of the LTB fusion protein in the SARS-CoV-2 construct increased the amount of high molecular weight complexes. Experiments in mice will directly compare mucosal IgA and neutralizing antibody responses after vaccination with the maize-produced S1 protein alone and S1 fused to LTB, upon challenge with both the original Wuhan-Hu-1 strain as well as the most relevant current circulating strain.

While a simple oral administration is preferable, the expression of recombinant protein in maize is also amenable to the production of large amounts of antigens for more traditional parenterally administered subunit vaccines. In conclusion, we demonstrated that the spike protein from an early strain of SARS CoV-2 can be produced at high levels in maize. We have also shown that for both SARS CoV-2 and PEDV, the S1:LTB fusion protein can enhance the formation of higher molecular weight complexes, and in the case of PEDV, increase the mucosal response in pigs. This represents a significant step toward the reliable implementation of practical low-cost vaccines produced in maize. Whether for antigen production for traditional injected subunit vaccines or ideally for simple oral administration, the results are encouraging for further development of this system for veterinary use, as a booster to address new variants in the current COVID-19 pandemic, and to address future emerging pathogens. Although further studies are needed to fully characterize this observation, the work described here presents a novel finding on how antigen fusion to LTB for coronaviruses, particularly in maize-produced vaccines, can increase efficacy.

## Figures and Tables

**Table 1:** Pig study treatment groups.

Group	Treatment	Primary	Boost 1 (Day 21)	Boost 2 (Day 35)	Boost 3 (Day 49)
INJ	Positive control	Inject	Inject	Inject	Inject
CON	Negative control	Inject	Control corn	Control corn	Control corn
PDC	Oral vaccine: PDC	Inject	Oral PDC	Oral PDC	Oral PDC
PDK	Oral vaccine: PDK	Inject	Oral PDK	Oral PDK	Oral PDK

## Data Availability

Data are contained within the article and additional details and materials are available upon request from the authors.
